# The influence of 8,786 Western China kindergarten teachers' emotional intelligence on work engagement

**DOI:** 10.3389/fpsyg.2025.1542911

**Published:** 2025-03-25

**Authors:** Zedong Zhang, Yan Li, Ye Wang, Xiaomin An

**Affiliations:** ^1^Faculty of Education, Northeast Normal University, Changchun, China; ^2^Department of Teacher Education, Xilingol Vocational College, Xilinhot, China; ^3^School of Preschool Education, Changchun Normal College, Changchun, China; ^4^China Rural Education Development Institute, Northeast Normal University, Changchun, China

**Keywords:** correlation, education background, emotional intelligence, kindergarten teacher, online survey, work engagement

## Abstract

Although emotional intelligence (EI) has been shown to influence work engagement significantly (WE) in many professions, the relationship between EI and WE among kindergarten teachers in Western China remains underexplored. This study aims to fill this gap by investigating how the EI of kindergarten teachers affects their WE and whether educational background moderates this relationship. A sample of 8,786 kindergarten teachers completed the Emotional Intelligence Scale for Kindergarten Teachers and the Work Engagement Scale. The results indicate that EI is positively correlated with and predictive of WE among kindergarten teachers. However, within the internal structure of EI, emotional perception does not predict WE, while emotional identification negatively predicts it. Furthermore, the educational background of kindergarten teachers was found to moderate the relationship between EI and WE. To enhance WE among kindergarten teachers in the future, it is crucial to view EI as a developable skill. This can be accomplished by providing teachers with diverse social practice opportunities and offering structured EI training programs.

## 1 Introduction

As the first significant others encountered by children when they transition from the family to a broader social environment, kindergarten teachers play a crucial role in influencing both the immediate wellbeing and long-term development of young children (Soininen et al., 2023). The ability of teachers to engage passionately in their work is vital, and emotional states are a primary internal factor affecting a teacher's WE. Emotions are a core aspect of both their professional responsibilities and personal lives (Schutz and Zembylas, 2009). Therefore, enhancing the EI of kindergarten teachers is particularly critical for improving their WE.

### 1.1 Work engagement

In 1990, Kahn formally introduced the concept of WE and delineated its dimensions, defining it as “the harnessing of organizational members' selves to their work roles”. He categorized engagement into three types: physical, cognitive, and emotional (Kahn, 1990; Schaufeli et al., 2002). According to Kahn, whether working alone or with others, individuals invest physically in their work, expressing their thoughts, feelings, creativity, beliefs, and values. This investment results in cognitive alertness and empathy toward others.

In 1997, Maslach offered a different understanding of engagement and burnout as a continuous and unified whole (Maslach et al., 1986). In this framework, WE is composed of three dimensions: energy, involvement, and efficacy, each of which contrasts with the corresponding dimensions of burnout. Individuals experiencing high levels of burnout often feel ineffective, depleted, and disconnected from their work and colleagues. Conversely, those with high engagement are highly motivated, exhibit strong work efficiency, receive better compensation, maintain positive interpersonal relationships, and are more adaptable to work-related challenges. Maslach conceptualized the work state as a continuum, with burnout at one end and engagement at the other, allowing for various degrees of burnout and engagement in between.

In 2001, Britt proposed that WE consists of three dimensions: responsibility, commitment, and perceptions of the impact on performance (Britt et al., 2001). His study was based on the three-dimensional responsibility model introduced by Schlenker in 1994, focusing primarily on soldiers in peacekeeping forces to understand their sense of duty in fulfilling their mission and the significance of being a good soldier. Britt argued that WE reflects a strong sense of personal responsibility and a willingness to commit to one's performance, with the quality of that performance being closely tied to the individual's identity.

In 2002, Schaufeli proposed that WE is a positive, work-related emotional state characterized by vigor, dedication, and concentration (Schaufeli et al., 2002). This state is marked by persistence and dispersion rather than being directed toward a specific goal, event, or situation.

In 2004, May, Gilson, and Harter expanded on this idea by arguing that WE comprises physical, affective, and cognitive components (May et al., 2004). This dimensional framework aligns closely with the physical, cognitive, and emotional components originally proposed by Kahn. In 2010, Rich built upon Kahn's three-dimensional theoretical model, using it as a foundation to explore the factors that influence employee WE (Rich et al., 2010).

The dimensional categorization of WE has varied across studies, but it has been examined and understood from multiple perspectives and dimensions. Since Kahn introduced the concept of WE in 1990, it has attracted attention from various disciplines, including education, psychology, and organizational behavior. Research indicates that WE, as a positive psychological state, can have beneficial effects on organizations and reduce employees' propensity to leave their jobs (Saks, 2006; Culibrk et al., 2018; Nabhan and Munajat, 2023). Schaufeli further developed a model of WE and designed the Utrecht Work Engagement Scale (UWES), which explicitly includes the dimensions of vigor, dedication, and absorption. The vigor dimension comprises six items, the dedication dimension includes five items, and the absorption dimension contains six items, with the correlation coefficients among these three factors all exceeding 0.65 (Schaufeli et al., 2006).

Due to its crucial role in predicting individual performance and organizational effectiveness, along with the stability and scientific validity of its measurement, WE has become a prominent topic across various disciplines and fields in recent years. Research on the factors influencing WE has progressively expanded. The study of antecedent variables for WE can be broadly categorized into two dimensions: internal factors within the individual and external factors outside the individual.

Research has shown that physiological factors significantly influence WE. For instance, some studies have found that men tend to have higher levels of WE than women (Watkins et al., 1991), Age is positively correlated with WE, while chronic poor sleep quality has been shown to reduce it (Barber et al., 2012). Psychological factors also play a crucial role; personality toughness, for example, is significantly positively correlated with WE (Britt et al., 2001). Different psychological dispositions, such as low negativity and high enthusiasm, are strongly associated with better individual performance and higher engagement in work. EI, particularly emotional comprehension, has been found to positively correlate with WE, enhancing individuals' dedication to their roles (Durán et al., 2004; Ravichandran et al., 2011). Furthermore, there is a significant positive correlation between kindergarten teachers' sense of dignity and their WE (Bühler and Land, 2003; Gustafsson et al., 2009).

External factors also play a significant role in influencing WE. Organizational support, for example, has been found to be significantly and positively correlated with WE (Boikanyo and Naidoo, 2023). While social support is a less potent predictor, it still positively influences WE (Fouad et al., 2012). In the context of preschool education, extensive research has been conducted on how external factors affect kindergarten teachers' WE. Studies indicate that both career satisfaction and job satisfaction are strong predictors of teacher WE (Timms and Brough, 2013; Zang and Feng, 2023). Moreover, organizational identification is significantly and positively related to kindergarten teachers' WE (Ji and Cui, 2021). Despite these findings, there is still a notable gap in research focusing on the internal subjective factors that contribute to kindergarten teachers' WE.

Kindergarten teachers' commitment to their work is primarily reflected in their physical and emotional involvement, with their primary focus being on preschool children (Lestari et al., 2021). This commitment directly impacts the young children they teach. Given that preschool children are highly imitative, they are particularly sensitive to the words and actions of their teachers (Gergely and Csibra, 2005). The emotions, whether positive or negative, expressed by teachers are easily perceived by children and can significantly influence their psychological development (Hargreaves, 2000). Therefore, it is essential to prioritize the engagement levels of early childhood educators to foster a positive and supportive learning environment for young children.

### 1.2 Emotional intelligence

Internal factors, such as individual traits and EI, have been significant topics of debate in both theory and practice for over three decades. This controversy primarily arises from two perspectives: the academic viewpoint, led by Mayer and Salovey, who categorize EI as a form of intelligence and advocate for its formal integration into the field of psychology, and the pragmatic viewpoint, represented by Goleman, who emphasizes essential factors for predicting success beyond traditional intelligence measures. Mayer and Salovey defined EI as a mental ability related to cognitive processes, including the capacity to perceive and express emotions, facilitate thinking through emotions, understand emotions, and regulate them (Mayer and Salovey, 2000). They developed the MSCEIT.V scale, although it has faced criticism due to issues with low internal consistency reliability indicators. In contrast, Goleman conceptualized EI as comprising emotional awareness, emotional regulation, self-motivation, affective transference, and interpersonal relationships (Goleman et al., 2002).

Research has laid the groundwork for addressing theoretical issues related to EI. Despite ongoing debates about its definition, structure, and measurement, the field of EI continues to grow in significance. Studies have shown that as the emotional labor required in a job increases, the predictive power of an individual's EI on job satisfaction and performance also rises (Wong and Law, 2002). Kindergarten teachers, in particular, engage in more emotional labor compared to other occupational groups. Moreover, the professional stigmatization of kindergarten teachers has intensified in recent years (Huang et al., 2022). Consequently, enhancing the overall work performance of kindergarten teachers from their perspective has become a critical issue that demands attention.

From the perspective of early childhood education, experiencing and managing emotions is a fundamental aspect of an early childhood teacher's role and a critical component of EI. Early childhood teachers interact with a diverse group of young children throughout the day, requiring them to navigate and express a wide range of emotions, including happiness, sadness, anger, and shame. The influence of these emotions on their work can vary based on their level of EI, a concept encapsulated by emotional labor. Teachers with low EI are more likely to experience negative emotions such as low mood, fatigue, or even burnout (Fiorilli et al., 2019). Consequently, this can lead to adverse outcomes for the children, undermining the “child-centered” approach that is central to early childhood education.

### 1.3 Work engagement and emotional intelligence

While much has been studied about EI and WE in professions such as police officers, nurses, and special education teachers, research focusing on kindergarten teachers remains relatively sparse. In policing, higher emotional labor demands are associated with lower WE, as the need to suppress emotions and cope with traumatic events leads to emotional exhaustion, thereby diminishing officers' enthusiasm and dedication to their work (Oliveira et al., 2022). Similarly, EI has been shown to enhance occupational wellbeing and WE among Chinese clinical nurses (Gao et al., 2024). Special education teachers, too, benefit from higher EI, which leads to greater WE, as they are more dedicated, energetic, and absorbed in their roles. EI helps them manage the emotional demands of their work, reducing burnout and fostering greater engagement (Wang et al., 2024a).

Research has consistently found that the more emotional labor a profession demands, the stronger the predictive power of EI on job satisfaction and performance (Wong and Law, 2002). Among these professions, kindergarten teachers are required to engage in more emotional labor than most other occupational groups. The emotional labor involved in teaching young children is particularly demanding, as teachers must not only manage their own emotions but also regulate and respond to the emotions of their students. This makes EI especially crucial for fostering WE in early childhood education. Despite its importance, studies specifically focusing on kindergarten teachers remain limited.

Previous studies have primarily analyzed demographic variables (Wang, 2022), revealing differences in EI performance between genders, with females showing higher emotional perception and males demonstrating better emotional recovery (Sanabrias-Moreno et al., 2023). However, few studies have examined demographic variables as moderating effects. Demographic variables, particularly educational background, are internal factors that warrant attention. Education background, which can be enhanced through personal effort, may influence EI (Vahedi and Nikdel, 2011). Therefore, this study aims to explore whether kindergarten teachers' EI affects their WE and whether education background moderates this relationship.

### 1.4 The study

While EI is recognized as important in professions such as nursing and policing, there is limited research on its impact in early childhood education. Given the emotional demands of their work, it is crucial to understand how EI influences kindergarten teachers' engagement with their roles. Additionally, the role of educational background, as a demographic factor, has been largely unexplored in this context. So this study examines the relationship between EI and WE among kindergarten teachers and investigates whether educational background moderates this relationship.

This study proposes the following three hypotheses based on a review of the literature on EI and WE:

Hypotheses 1 (H1): EI is positively correlated with the WE of kindergarten teachers.

Hypotheses 2 (H2): The dimensions of EI are correlated with the dimensions of WE.

Hypotheses 3 (H3): Education background moderates the relationship between EI and WE.

## 2 Materials and methods

### 2.1 Participants and procedures

This study utilized a convenience sampling method for both the questionnaire survey and interviews. Data were collected from currently employed kindergarten teachers in a province in Western China. The questionnaire survey was administered online, while the interviews were conducted through a combination of online and offline methods. The aim was to explore issues not fully addressed by the questionnaire alone, focusing on kindergarten teachers from various backgrounds. A total of 10,141 questionnaires were distributed over a two-week period. After excluding invalid responses, 8,786 valid questionnaires were returned, yielding a validity rate of 87%.

Among the respondents, 265 were male (3.0%) and 8,521 were female (97.0%). Regarding marital status, 6,343 were married (72.2%) and 2,443 were single (27.8%). The types of kindergartens were public for 5,659 (64.4%), private for public welfare for 2,437 (27.7%), and private non-public welfare for 690 (7.9%). Educational backgrounds included high school/vocational school or below for 1,565 respondents (17.8%), associate degree for 3,262 (37.1%), bachelor's degree for 3,893 (44.3%), and master's degree or above for 66 (0.8%).

### 2.2 Measures

#### 2.2.1 Emotional intelligence scale for kindergarten teachers

In this study, a self-developed Kindergarten Teacher Emotional Intelligence Scale was used. The scale's dimensions were based on a rational reflection on the concept of emotional intelligence, with specific items referencing WLEIS and EIS (Schutte et al., 1998; Wong and Law, 2002), while considering the specific context of this study. The initial scale comprised 49 items. Following expert evaluation and small-scale pre-testing, the scale underwent item analysis, exploratory factor analysis, and reliability and validity tests. The scale was scored using a five-point Likert scale, with responses categorized as “strongly disagree,” “disagree,” “unsure,” “agree,” and “strongly agree.” The overall reliability of the scale was above 0.90, with individual dimensions demonstrating reliabilities ranging from 0.89 to 0.93. These results confirm the scale's suitability as a reliable research tool.

As a result, a formal questionnaire was established, consisting of 33 items across four dimensions.

Emotion Perception Dimension includes 8 items that describe the basic ability to recognize one's own or others' emotional states. For example, “I can feel my friends' emotions from their behavior.”

Emotion Understanding Dimension consists of 6 items that focus on understanding the reasons behind emotional changes and their outcomes. For instance, “When I am in a bad mood, I can tell why.”

Emotion Identification Dimension includes seven items related to distinguishing between different emotional activities and complex emotional terminology. An example item is, “I can differentiate between 'fear' and 'panic.”'

Emotion Management Dimension contains 12 items that describe the application of perceived, understood and labeled emotional information to guide behavior and interactions. It involves forming an emotionally inclusive mindset and effective planning and execution. For instance, “When my friend is depressed, I can help him shift his attention to something happy.”

#### 2.2.2 Work engagement scale

The Work Engagement Scale, developed by Schaufeli in 2002, comprises three dimensions and a total of 17 items (Schaufeli et al., 2002). 6 items in the vitality dimension, describing an individual's willingness to work hard for his/her job, and his/her ability to be energetic and resilient at work, and to persevere when encountering difficulties, such as “I feel strong and energetic at work”; 5 items in the devotion dimension, describing an individual's ability to be passionately and wholeheartedly engaged in his/her work, and to experience a sense of meaning and honor at work, and to accept challenges encountered at work; 5 items in the dedication dimension, describing individuals who are able to devote themselves to their work with enthusiasm, experience a sense of meaning and honor in their job, and accept the challenges they encounter in their work, such as “I am passionate about my work”; 5 items in the concentration dimension, describing the individual's ability to concentrate on his/her work and to be happily immersed in it, and his/her unwillingness to detach himself/herself from his/her work, such as “I forget everything around me when I am working”. The scale is based on a 5-point scale, with the higher the score, the higher the level of WE. The scale uses a 5-point Likert scale, with higher scores indicating higher levels of WE. The scale is scored using a 5-point Likert scale, with higher scores indicating higher levels of WE. This scale is well-established and widely used across various fields and countries. In this study, the reliabilities of the dimensions—vigor, dedication, and absorption—ranged from 0.90 to 0.92, confirming its effectiveness as a research tool.

#### 2.2.3 Demographic characteristic questionnaire

One demographic characteristic collected via a self-designed questionnaire which includes gender (male, female), marital status (married, single), educational backgrounds (high school/vocational school or below, associate degree, bachelor's degree, master's degree or above), types of kindergarten (public, private for public welfare, private non-public welfare).

### 2.3 Data analysis

Data analysis was conducted using SPSS 26.00. After data cleaning, which involved checking for missing values and outliers, descriptive statistics (mean and standard deviation) were calculated for EI and WE variables. Prior to conducting statistical tests, we verified that the data met the assumptions of normality, linearity, and homoscedasticity. A multiple linear regression analysis was then conducted to investigate the impact of EI and its subdimensions on WE. This analysis enabled us to assess the direct relationships between EI and WE while controlling for other variables. To further explore whether educational background moderates the relationship between EI and WE, hierarchical regression analysis was employed. Additionally, the PROCESS 4.1 plug-in was utilized for a more detailed analysis of the moderating effect of educational background, allowing us to examine how varying levels of educational attainment influence the strength of the relationship between EI and WE, based on conditional effects at different moderator levels.

### 2.4 Research ethics

In this study, written informed consent was obtained from each participant. All collected data were kept anonymous and confidential to protect participants' privacy. Additionally, ethical approval was granted by the Ethics Committee of Northeast Normal University.

## 3 Results

### 3.1 Descriptive statistical analysis of kindergarten teachers' emotional intelligence and work engagement

As shown in Table 1, the EI scores of kindergarten teachers are as follows: overall EI is 4.07, with emotional understanding at 4.26 and emotional management at 3.98. The WE scores are: overall WE at 3.90, dedication at 4.04, and vigor at 3.79.

**Table 1:** Means, standard deviations, and correlations of the major variables.

	**1**	**2**	**3**	**4**	**5**	**6**	**7**	**8**	**9**
1. Emotional perception	1								
2.Emotional understanding	0.66^**^	1							
3.Emotional identification	0.70^**^	0.72^**^	1						
4.Emotional management	0.63^**^	0.68^**^	0.65^**^	1					
5.Vigor	0.33^**^	0.36^**^	0.33^**^	0.57^**^	1				
6.Dedication	0.36^**^	0.42^**^	0.37^**^	0.57^**^	0.79^**^	1			
7.Absorption	0.34^**^	0.38^**^	0.35^**^	0.55^**^	0.79^**^	0.81^**^	1		
8.EI	0.85^**^	0.85^**^	0.86^**^	0.90^**^	0.48^**^	0.52^**^	0.49^**^	1	
9.WE	0.37^**^	0.41^**^	0.37^**^	0.60^**^	0.93^**^	0.92^**^	0.93^**^	0.53^**^	1
Mean average (*SD*)	4.04 (0.47)	4.26 (0.52)	4.09 (0.54)	3.98 (0.52)	3.79 (0.68)	4.04 (0.64)	3.90 (0.67)	4.07 (0.47)	3.90 (0.62)

^**^p < 0.01.

The analysis revealed a significant positive correlation between kindergarten teachers' EI and their WE (*p* < 0.01), both overall and within individual dimensions (*p* < 0.01). Among these dimensions, emotional management demonstrated a particularly strong correlation with vigor, dedication, and concentration compared to other dimensions. These findings support H2, which posits that the dimensions of EI are correlated with the dimensions of WE.

### 3.2 Regression analysis of kindergarten teachers' emotional intelligence and work engagement

We conducted a multiple linear regression analysis to examine the impact of EI and its dimensions on WE. The results, presented in Table 2, indicated a positive effect of EI on WE, with an F-value of 3,443.69 and a *p*-value of 0.00. This significant F-value for the overall regression model suggests that EI is a highly significant predictor of WE, with at least one of its subdimensions serving as a significant predictor. These findings confirm H1, which posits that kindergarten teachers' EI is positively correlated with WE.

**Table 2:** Regression analysis of EI and WE.

	***B***	**β**	***t***	***R^2^***	***F***	**VIF**
EI	0.72	0.01	58.68^***^	0.28	3,443.69^***^	1.00
Emotional perception	−0.02	0.02	−1.19	0.36	1,250.16^***^	2.29
Emotional understanding	0.03	0.02	2.12^*^			2.59
Emotional identification	−0.04	0.02	−2.63^*^			2.63
Emotional management	0.73	0.02	48.64^***^			2.20

^*^p < 0.05; ^***^p < 0.001.

The analysis revealed that emotional perception (*t* = −1.19, *p* > 0.05) did not significantly predict WE among the sub-dimensions. However, emotional understanding, identification, and management all had significance levels below 0.05, indicating a significant relationship with WE. Notably, emotional identification was identified as a negative predictor.

Hierarchical regression analysis was used to explore the potential moderating effect of educational attainment on the relationship between EI and WE. Educational attainment, a multi-categorical variable, was converted into dummy variables, with “High School/Secondary School and Below” serving as the reference category in the regression analyses.

The results, presented in Table 3, indicate that neither “Junior College” (β = −0.01, *p* > 0.05) nor “Undergraduate” (β = −0.01, *p* > 0.05) moderated the relationship between EI and WE. However, having a “Master's Degree or Above” negatively moderated this relationship compared to the reference category, with a β value of −0.02 (*p* < 0.001). These findings support H3, which posits that educational background moderates the relationship between EI and WE.

**Table 3:** Summary of hierarchical regression analysis for variables predicting WE.

**Variable**	***B***	***SE***	**β**	***t***	***R^2^***
**Model 1**					
EI	0.73	0.01	0.54	60.92^***^	0.32
Junior college	−0.16	0.02	−0.12	−9.89^***^	
Undergraduate	−0.33	0.02	−0.26	−21.55^***^	
Master degree or above	−0.52	0.06	−0.07	−8.06^***^	
**Model 2**					
EI × Junior college	−0.03	0.04	−0.01	−0.73	0.32
EI × Undergraduate	−0.02	0.04	−0.01	−0.43	
EI × Master degree or above	−0.33	0.12	−0.02	−2.84^**^	

^**^p < 0.01; ^***^p < 0.001.

**Figure 1:**
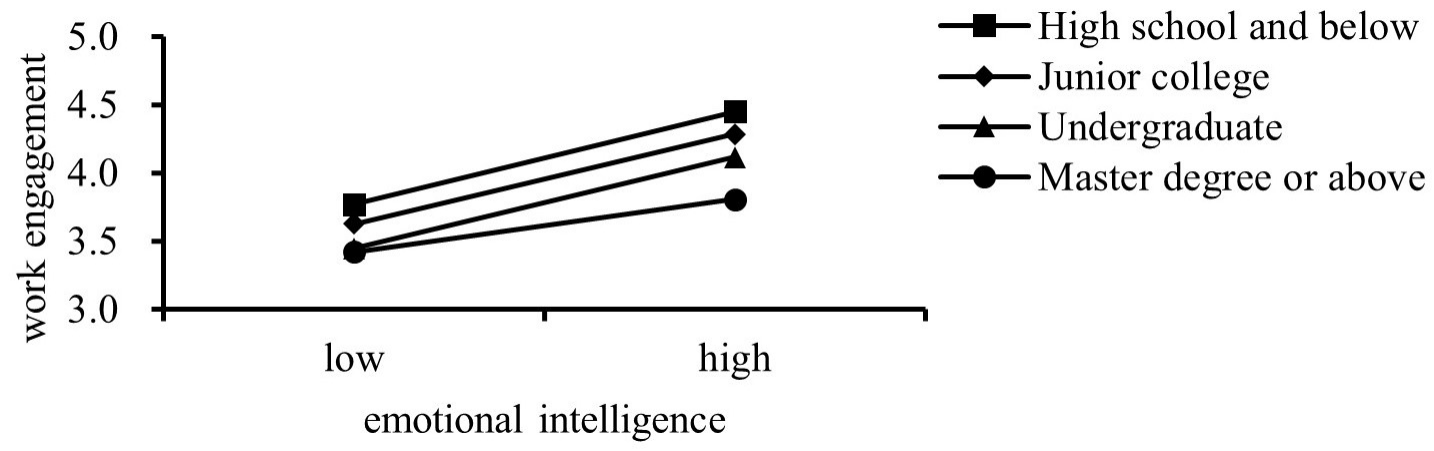
The moderating effect of education background on EI and WE.

Further analysis was conducted using the PROCESS 4.1 plug-in to test the moderating effect of educational background on the relationship between EI and WE. Simple slope plots were generated to visualize this interaction. The results, detailed in Table 4 and illustrated in Figure 1, show that educational background significantly moderates the relationship between EI and WE. This is evidenced by a notable change in R2, indicating a significant moderating effect.

**Table 4:** Test of the moderating effect of education background.

	***R^2^***	***F***	***df_1_***	***df_2_***	***p***
Education background	0.00	2.77	3.00	8,778.00	0.04

## 4 Discussion

Previous research has often overlooked the specific relationship between EI and WE among kindergarten teachers. This study's findings reveal that kindergarten teachers with higher levels of education tend to have greater capabilities in emotional perception, emotional understanding, and emotional identification. In contrast, those with lower levels of education exhibit greater proficiency in emotional management. This suggests that higher educational attainment does not necessarily equate to superior emotional management skills among kindergarten teachers.

### 4.1 Performance on kindergarten teachers' emotional intelligence and work engagement

#### 4.1.1 General performance

The results of the analysis indicate that kindergarten teachers in this survey exhibit high levels of EI and moderately high WE. Overall, both kindergarten teachers' EI and WE levels were relatively high, which is consistent with previous research findings (Poulou, 2017; Wang et al., 2024b). On one hand, this could be due to the relatively strong economic development, rich historical and cultural heritage, and high level of educational development in the selected province in Western China. On the other hand, it may also be attributed to the Chinese government's continuous efforts over the years to support educational development in the western regions, optimizing the environment for early childhood education and enhancing the attractiveness of the teaching profession.

#### 4.1.2 Specific performance

In terms of EI, kindergarten teachers' scores for emotional management are lower compared to their scores for emotional perception, emotional understanding, and emotional identification. This finding aligns with a previous study by Martyniak and Pellitteri (2020). Emotional management, being the most advanced aspect of EI, involves applying and refining accumulated emotional experience through continuous practice.

Additionally, kindergarten teachers' emotional management is significantly affected by practical concerns, particularly those related to non-teaching activities. Tasks such as interacting with parents have been reported to negatively impact their mood. As highlighted in interviews, teachers voiced frustrations such as: “There are many chores in the workplace beyond teaching activities that disturb me. I feel that these tasks, like various daily inspections and paperwork, affect my mood. There are many things other than care and education activities that need to be done.” Another teacher noted, “Dealing with parents is a challenging aspect of the job. After many years, I still encounter parents who do not cooperate, neglect their child's growth, exaggerate issues, or escalate complaints to higher authorities, which makes it difficult to manage these situations.”

Regarding WE, kindergarten teachers exhibit high levels of dedication but relatively lower vigor (Sun et al., 2025). Interviews reveal that teachers generally have a strong job commitment, enjoy their work, and experience positive working conditions. For example, one teacher mentioned, “I find working in a kindergarten quite fulfilling, and my direct experience is that I am happier with the children. If given another choice, I would still pursue this profession.” This reflects that despite facing numerous challenges, kindergarten teachers remain devoted to their work and derive a sense of achievement and satisfaction from their role. Regarding WE, kindergarten teachers with low educational levels are more engaged than those with high (Jeon et al., 2018). As preschool education evolves, many kindergarten teachers with higher educational qualifications are being added to the workforce. Some with postgraduate degrees are now part of the teaching staff, significantly impacting existing lower-educated teachers. Lower-educated teachers, concerned about job security amid these changes, have increased their efforts and commitment to their roles.

### 4.2 The relationship between kindergarten teachers' emotional intelligence and work engagement

EI showed a significant positive correlation with overall WE, which is consistent with findings from previous research indicating that emotional management has the most substantial correlation with various dimensions of WE (George et al., 2022). This may be attributed to the fact that emotional management, as an external dimension, is more readily expressed through verbal behavior, unlike the more implicit dimensions of emotional perception, understanding, and identification.

#### 4.2.1 Emotional intelligence positively predicts work engagement

Kindergarten teachers' EI predicts WE. A research on nurses similarly found that EI significantly enhances WE, contributing to reduced nurse turnover, improved patient outcomes, and maintaining a competitive edge in healthcare settings (Al Otaibi et al., 2023; Gao et al., 2024). Although research specifically on kindergarten teachers is limited, the complexity of their work requires a substantial emotional investment to ensure the quality of early childhood education. Additionally, emotional understanding and emotional management are key predictors of WE. The ability to accurately recognize and be aware of emotions allows individuals to manage and control them effectively, fostering positive attitudes and behaviors in the workplace (Antonopoulou, 2024).

#### 4.2.2 Emotional perceptions do not predict work engagement

Previous studies have not thoroughly examined the impact of the sub-dimensions of EI, particularly the role of emotional perception in predicting WE. In this study, emotional perception did not predict WE, a finding consistent with some prior research (De Clercq et al., 2014; Zhu et al., 2015). This result may be attributed to the fact that while individuals can recognize their emotions and those of others, this basic level of awareness does not necessarily translate into effective emotional management or application in the workplace. The transition from knowing to doing requires a deeper understanding and practical application of emotions, which necessitates continuous practice and development. Therefore, mere awareness of emotions does not substantially impact actual work performance.

#### 4.2.3 Emotional identification negatively predicts work engagement

Regarding the sub-dimension of EI and emotional identification, the present study's findings were contrary to the initial hypothesis for two reasons. Firstly, while emotional identification involves the ability to differentiate and recognize complex emotions more clearly, it does not necessarily translate into increased WE. In some cases, being able to identify emotions may not directly enhance work performance; instead, it might lead to emotional confusion that does not impact work motivation. Secondly, during interviews, respondents struggled to accurately answer questions about emotional identification. This suggests that some teachers may respond to questionnaires based on social desirability rather than their actual experiences (Krumpal, 2023). Consequently, this highlights the need for further refinement in measuring this dimension of EI.

### 4.3 Moderating effects of education background

From a sociological perspective, demographic factors influence individual values, personality, temperament, and abilities, which in turn affect emotions and WE, as demonstrated in previous research (Wang, 2022; Zang and Feng, 2023; Huang and Zhou, 2024). In the present study, educational background was found to significantly and positively moderate the relationship between EI and WE. Specifically, kindergarten teachers with a master's degree showed a stronger positive moderation between EI and WE.

This may be attributed to the fact that highly educated kindergarten teachers, having undergone more extensive professional training and accumulated a greater body of theoretical knowledge, are better equipped to regulate their EI and WE. The influx of such highly educated teachers into kindergartens can also serve as a catalyst for teachers with lower educational backgrounds to enhance their competitiveness.

The moderating effect of educational background highlights the importance of considering demographic variables when examining the relationship between EI and WE. It underscores the need to tailor training programs to address the diverse needs of teachers at different educational levels, and to further explore how various demographic factors influence work-related outcomes.

### 4.4 Implications

From a theoretical perspective, the study contributes to the growing body of research on EI and WE in early childhood education. From a practical standpoint, this study underscores the importance of EI in enhancing the WE of kindergarten teachers. Practically, it highlights the critical role EI plays in fostering greater work engagement among kindergarten teachers. Educational institutions and policymakers should prioritize the implementation of targeted training programs aimed at enhancing EI. These programs should focus on strategies such as emotional regulation, empathy, and effective communication, which not only promote teachers' emotional wellbeing but also enhance their engagement with their work.

### 4.5 Limitations and future directions

While this study provides valuable insights into the relationship between EI and WE among kindergarten teachers, several limitations should be acknowledged. First, the cross-sectional design limits causal inferences between EI and WE. Longitudinal studies are needed to establish a clearer cause-and-effect relationship. Second, the sample was drawn from a specific geographic area, which may limit the generalizability of the findings to other regions or cultural contexts. Future research could explore a more diverse sample across different regions or countries. Lastly, the role of other demographic variables, such as teaching experience or school type, could be explored to provide a more comprehensive understanding of the factors influencing WE in early childhood education.

## 5 Conclusions

The study found that kindergarten teachers in a province in western China exhibited a moderately high level of WE, and EI was significantly positively correlated with WE. Within the internal structure of EI, emotional perception did not predict WE, while emotional identification negatively predicted it. Additionally, education background was found to positively moderate the relationship between EI and WE.

These results underscore the importance of valuing EI in professions that require substantial emotional labor. For kindergarten teachers, enhancing EI through upgrading educational qualifications can lead to increased WE and overall effectiveness in their roles.

## Data Availability

The raw data supporting the conclusions of this article will be made available by the authors, without undue reservation.
