# Supplementary material for: The influence of 8,786 Western China kindergarten teachers' emotional intelligence on work engagement
Source: Front Psychol. 2025 Mar 25;16:1542911. doi: 10.3389/fpsyg.2025.1542911 (PMC11977667; doi:10.3389/fpsyg.2025.1542911)
Supplement: Supplementary file 1 [file Table_1.docx]

### Emotional Intelligence Scale for Kindergarten Teachers

This scale consists of 33 items (Likert-type scale). The emotional perception dimension includes items 1-8, the emotional understanding dimension includes items 9-14, the emotional identification dimension includes items 15-21, and the emotional management dimension includes items 22-33.

| Items | Strongly disagree | Disagree | Unsure | Agree | Strongly agree |
| --- | --- | --- | --- | --- | --- |
| 1. I can understand my own feelings. |  |  |  |  |  |
| 1. I can sense my friends' emotions from their behavior. |  |  |  |  |  |
| 1. I can recognize others' emotions by observing their expressions. |  |  |  |  |  |
| 1. I can perceive others' feelings and emotions. |  |  |  |  |  |
| 1. I understand the emotions of those around me. |  |  |  |  |  |
| 1. I can identify the person who is feeling down in a group. |  |  |  |  |  |
| 1. When talking on the phone, I can judge the caller's mood from their voice. |  |  |  |  |  |
| 1. I can determine a person's mood from their tone of speech. |  |  |  |  |  |
| 1. I know why I feel happy. |  |  |  |  |  |
| 1. When I feel unhappy, I can identify the reason behind it. |  |  |  |  |  |
| 1. I can recognize what causes my fear. |  |  |  |  |  |
| 1. I can understand why I experience certain emotions. |  |  |  |  |  |
| 1. I can assess whether my emotional state is conducive to work. |  |  |  |  |  |
| 1. I can predict the impact of prolonged negative emotions. |  |  |  |  |  |
| 1. I can distinguish between euphoria and joy. |  |  |  |  |  |
| 1. I can differentiate between fear and terror. |  |  |  |  |  |
| 1. I can distinguish between fear and panic. |  |  |  |  |  |
| 1. I can differentiate between jealousy and envy. |  |  |  |  |  |
| 1. I believe guilt and shame are different. |  |  |  |  |  |
| 1. I can distinguish between mania and restlessness. |  |  |  |  |  |
| 1. I think anxiety is a stronger emotional experience than worry. |  |  |  |  |  |
| 1. I can accept my emotional changes. |  |  |  |  |  |
| 1. I can accept the emotional changes of others. |  |  |  |  |  |
| 1. I can set goals for myself and strive to achieve them. |  |  |  |  |  |
| 1. When facing pressure, I can see it as motivation for success. |  |  |  |  |  |
| 1. Challenges inspire my creativity. |  |  |  |  |  |
| 1. When feeling disappointed, I can calmly reflect on my problems. |  |  |  |  |  |
| 1. When my friends are feeling down, I can help them shift their focus to happy things. |  |  |  |  |  |
| 1. When others are feeling depressed, I can help them feel better. |  |  |  |  |  |
| 1. When facing difficulties, I can control my temper. |  |  |  |  |  |
| 1. When I am angry, I can calm down quickly. |  |  |  |  |  |
| 1. I can respond appropriately to others' emotions. |  |  |  |  |  |
| 1. I can express my emotions and feelings smoothly. |  |  |  |  |  |
